# Case Report: Bloodstream infection due to *Clostridium innocuum* combined with *Eggerthella lenta*

**DOI:** 10.3389/fmed.2026.1747742

**Published:** 2026-01-28

**Authors:** Yan Xu, Anan Xu, Jiaying Du, Yu Zhao, Xiaoqing Liu, Zhewei Sun, Qingqing Xu, Manman Zhang, Yue-ru Tian

**Affiliations:** 1 Department of Laboratory Medicine, Huashan Hospital Fudan University, Shanghai, China; 2 Department of Laboratory Medicine, Liyang People's Hospital, Changzhou, China; 3 Department of Laboratory Medicine, Shidong Hospital, University of Shanghai for Science and Technology, Shanghai, China; 4 Key Laboratory of Clinical Pharmacology of Antibiotics, National Health Commission of the People's Republic of China, Beijing, China; 5 Department of Laboratory Medicine, Jinan Third People's Hospital, Jinan, China

**Keywords:** *Clostridium innocuum*, colorectal surgery, *Eggerthella lenta*, polymicrobial bloodstream infection, rectal cancer

## Abstract

**Background:**

The co-infection of *Clostridium innocuum* and *Eggerthella lenta* in bloodstream is uncommon. The choice of clinical drugs with which to treat such co-infection is limited, which is worthy of study.

**Case presentation:**

A 58-year-old male patient was admitted to the Department of General Surgery of Huashan Hospital on May, for further treatment after chemotherapy for liver metastases from rectal cancer. Laparoscopic anterior rectal resection under general anesthesia, laparoscopic sigmoid-rectal anastomosis, laparoscopic mesenteric lymphadenectomy, laparoscopic temporary ileostomy, and segmentectomy were performed. On the 3rd day after surgery, the patient developed a fever with body temperature up to 38 °C accompanied by cough and yellow sputum. Chest CT showed possible bilateral lung inflammation and metastases. Empirical anti-infection and symptomatic and supportive treatment were given. The patient still had a fever, with a body temperature of up to 40.6 °C, accompanied by fear of cold and chills, abdominal pain and abdominal distension on physical examination, abdominal wound non-healing, visible purulent secretions, and increased C-reactive protein, procalcitonin, and other inflammatory indicators. Aerobic and anaerobic blood culture tests were performed. The anaerobic blood culture bottle was positive after incubation in the automatic incubator for 20 h. After 24 h of anaerobic subculture on blood plate. *C. innocuum* was identified by matter-assisted laser desorption/ionization time of Flight (MALDI-TOF) mass spectrometry. After anaerobic culture time was extended to 72 h, another small slow-growing colony was observed, and *E. lenta* was identified. The patient’s postoperative history of rectal cancer showed the possibility of intestinal colonizing bacteria invading the bloodstream and causing infection. According to pieces of literature and drug sensitivity tests in our center, vancomycin 1 g + piperacillin / tazobactam 4.5 g were administered every 12 h for anti-infection for 7 days. The patient’s fever peak decreased, and blood culture turned negative after reexamination, thus the treatment was considered to be effective. Because the patient also had an abdominal infection and lung infection, antibiotic therapy with cefoperazone sulbactam and levofloxacin was continued for 9 days, and the patient had no further fever and was discharged with improved condition.

**Conclusion:**

*Clostridium innocuum and E. lenta* can cause bloodstream infection after colorectal surgery, and the above two rare anaerobic bacteria can be rapidly and economically identified by MALDI-TOF mass spectrometry. *C. innocuum, and E. lenta* isolated from bloodstream infections following colorectal surgery should be considered as pathogens and treated promptly and appropriately.

## Background

Bloodstream infection (BSI) refers to systemic inflammatory response syndrome caused by the invasion of pathogens and their toxins into the blood circulation. Blood culture, both aerobic and anaerobic, is the gold standard for diagnosing bloodstream infections (1). Anaerobic culture should be routinely performed when there are indications for blood culture, particularly recent gastrointestinal surgery, abdominal infection, immunosuppression, cancer, or other high-risk factors for anaerobic infection. In this paper, we present a rare case of polymicrobial bloodstream infection after colorectal surgery in a patient with liver metastases from rectal cancer, in which the positive cultures were identified as *C. innocuum* and *Eggerthella lenta*, respectively.

*Clostridium innocuum* (2) is an anaerobic gram-positive bacillus with 2–4 μm long and 0.4–1.01 μm wide, and oval spores can form at the end. The colonies on blood plates have a diameter of 1.5–2.0 mm and are white, smooth, convex, and without hemolysis (3). *C. innocuum*, first isolated from an appendiceal abscesses in 1962 by Smith and King (3), has been recognized as a commensal intestinal flora and a cause of rare opportunistic infections in immunodeficient patients (4). Chia et al. showed that *C. innocuum* was the second most common *Clostridium* species pathogen causing extraintestinal infections following *C. perfringens*, with a mortality rate of 16.7% for extraintestinal infections (5). Previous case reports have shown that *C. innocuum* can cause extraintestinal infections such as bacteremia, endocarditis, osteomyelitis, and peritonitis (6–8). Chia et al. confirmed the intestinal pathogenicity of *C. innocuum* by finding that it caused intestinal tissue edema, inflammation and necrosis in infected mouse models (9). *E. lenta* is also an obligate anaerobic gram-positive bacillus, non-spore-forming, and is characterized by slow growth and formation of small grayish colonies 0.25–0.75 mm in diameter after 72 h of anaerobic incubation. In 1935, Arnold Eggerth first reported that *E. lenta* as a commensal of human symbiont, which colonizes the intestinal tract, female reproductive tract and other parts of healthy humans, and is an opportunistic pathogen of appendicitis, liver abscess (10). *E. lenta* can cause bloodstream infections, gastrointestinal infections and soft tissue infections, with high susceptibility to empirical antibacterial drugs such as vancomycin, clindamycin, amoxicillin clavulanic acid, and carbapenems (10–12). 16S rRNA gene sequencing as well as MALDI-TOF mass spectrometry has enabled relatively precise identification of *C. innocua* and *E. lenta* (13, 14).

Among the pathogens causing bloodstream infections, anaerobic bacteria account for approximately 5% and associated mortality rates are approximately 15–50% (15). Mixed infections with anaerobic bacteria are even rarer and cause high mortality (16). Studies have shown that 30-day mortality is twice as high in mixed anaerobic bloodstream infections compared to single anaerobic bloodstream infections (17, 18). Therefore, for patients with positive anaerobic blood culture, early rapid and accurate diagnosis and timely use of appropriate drug therapy are particularly important. Acker et al. reported a rare case of mixed infection with four anaerobic bacteria including *E. lenta*, which was rapidly identified early by MALDI-TOF and empirically treated with meropenem combined with metronidazole against anaerobic bacteria to cure the patient, demonstrating the important clinical significance of bloodstream infection with multiple anaerobic pathogens and the importance of early and correct treatment (19). Bacteremia caused by a combination of multiple organisms, which represents translocation of microorganisms into the blood from the intestinal tract, skin, and mucosal surfaces, is relatively rare, accounting for only 4.7% of all sepsis (20, 21). In this paper, we present a case of bloodstream infection caused by *C. innocuum* and *E. lenta* after lower gastrointestinal surgery in a patient with rectal cancer, which has not yet been reported. In this case, these two anaerobic bacteria were rapidly and accurately identified by traditional isolation and culture, and gram staining combined with MALDI-TOF mass spectrometry. Timely anti-anaerobic treatment was given according to the results of drug susceptibility testing and relevant literature data, and finally the patient was discharged with stable vital signs. The clinical cure of bloodstream infection in this patient provides a reference for the treatment of bloodstream infection caused by multiple anaerobic bacteria.

## Case presentation

A 58-year-old male patient underwent laparoscopic anterior rectal resection, sigmoidorectal anastomosis, mesenteric lymph node dissection, laparoscopic temporary ileostomy, and segmental resection due to liver metastases after chemotherapy for rectal cancer. On the third day after surgery, the patient developed fever with a body temperature of 38 °C, yellow sputum, dyspnea, and blood oxygen saturation of 84.7%. Sputum culture showed *Enterobacter asburiae*, and chest CT showed possible bilateral lung inflammation and metastases. Combined with drug sensitivity results, empirical anti-infection and symptomatic and supportive treatment were given. Eleven days after surgery, the patient still had fever, with body temperature suddenly increased to 40.7 °C, accompanied by chills, abdominal pain and distension, non-healing abdominal wound, and purulent discharge. Chest CT showed inflammation in bilateral lower lobes with a small amount of bilateral pleural effusion and multiple scattered nodules in bilateral lung. Considering the possibility of metastases, abdominal CT showed postoperative changes of rectal cancer and changes in abdominal exudation. Blood work indicated whole blood C-reactive protein 71.57 mg/L, procalcitonin 31.39 ng/mL, IL-6 8.68 pg/mL, lactic acid concentration 3.24 mmol/L, oxygen saturation 63.4%, red blood cell count 3.28 × 10^12^/L, hemoglobin 104 g/L, white blood cell count 9.55 × 10^9^/L, neutrophils 93.7%, platelet count 101 × 10^9^/L, albumin 24 g/L, positive fecal occult blood, negative urine culture, wound secretion culture. Based on the above clinical manifestations and examination indicators, bloodstream infection was considered. Aerobic and anaerobic blood cultures were performed immediately, followed by return ileostomy, partial resection of small bowel, and debridement of abdominal wall wound were performed under general anesthesia.

**Figure 1:**
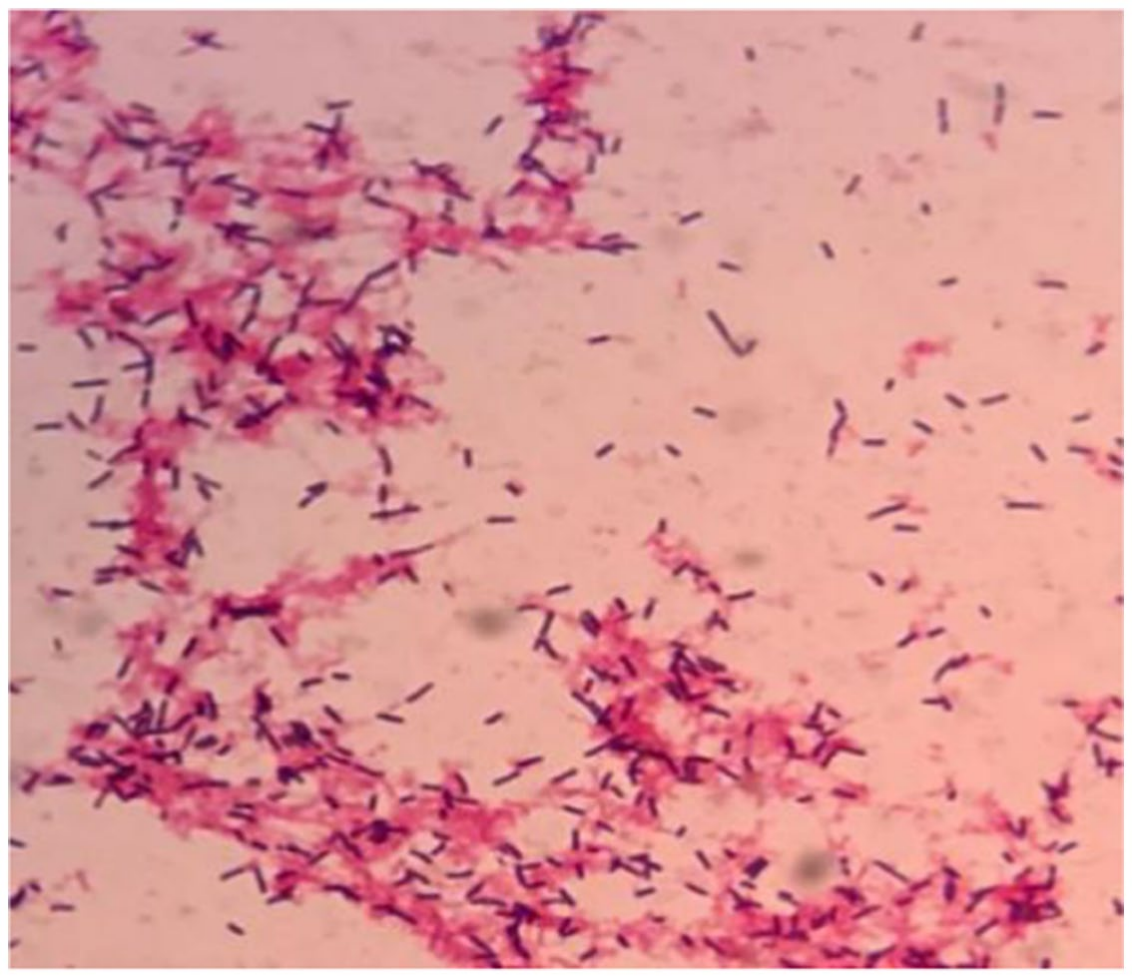
Twenty-hour positive curve of anaerobic blood culture bottle. Gram staining of direct smear of positive anaerobic blood culture bottle.

**Figure 2:**
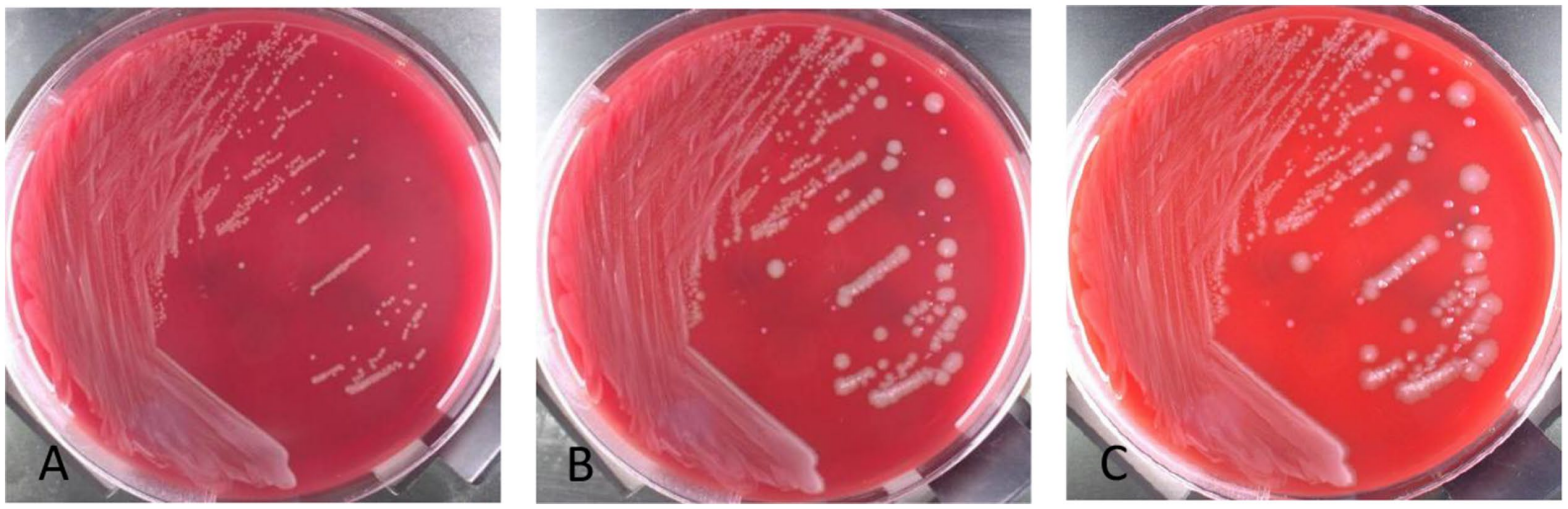
Colony morphology of positive anaerobic bottle transfer blood plate incubated anaerobically at 37 °C for different times. **(A)** Colony morphology after 24 h of subculture. **(B)** Colony morphology after 48 h of subculture. Rough, gray and large colonies were *C. innocuum*, and smooth, grayish white and small colonies were *E. lenta* exhibiting retarded growth. **(C)** Colony morphology after 72 h of subculture. Rough, gray and large colonies were *C. innocuum*, and smooth gray small colonies were *E. lenta* exhibiting retarded growth.

**Figure 3:**
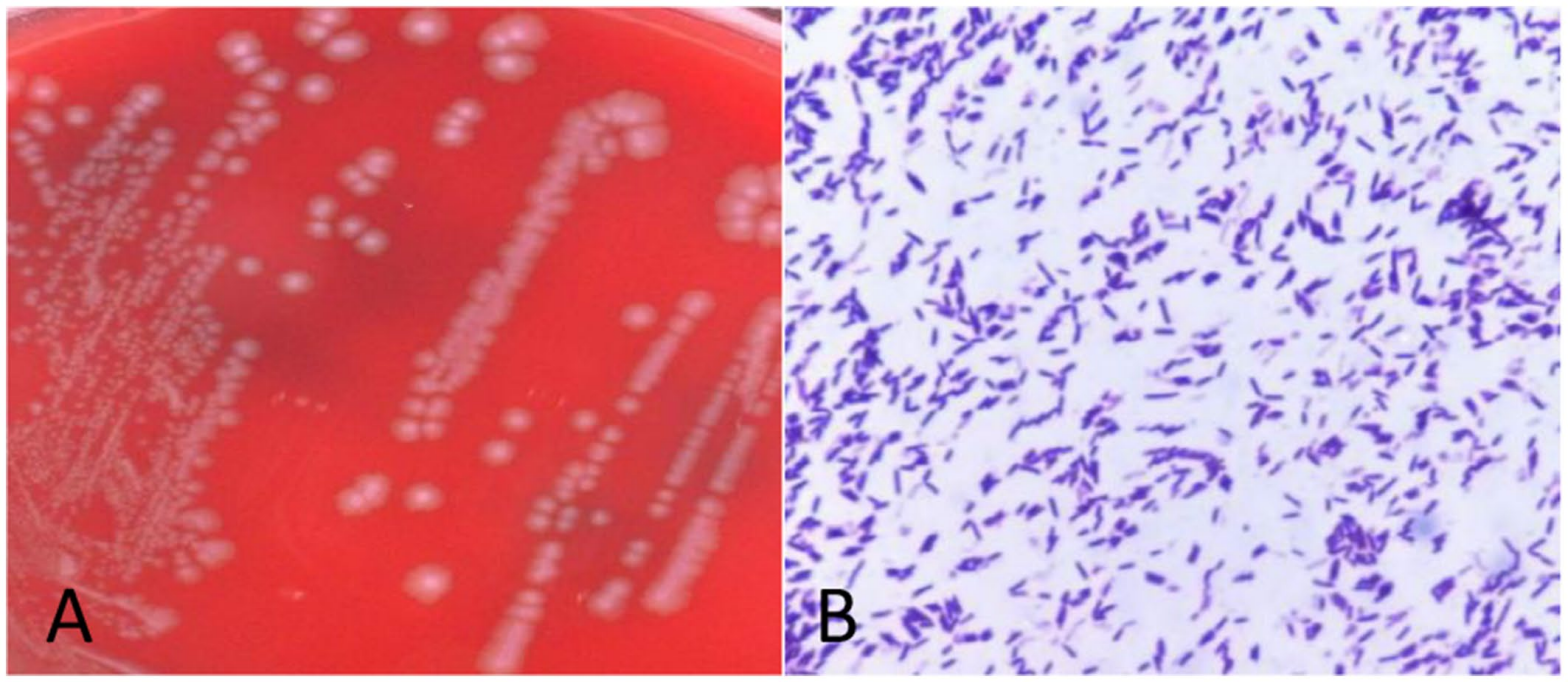
Morphology and gram staining of pure culture colonies of *Clostridium innocuum.* **(A)** Columbia blood plate pure division colony morphology. **(B)** Gram staining of single colony of *C. innocuum.*

**Figure 4:**
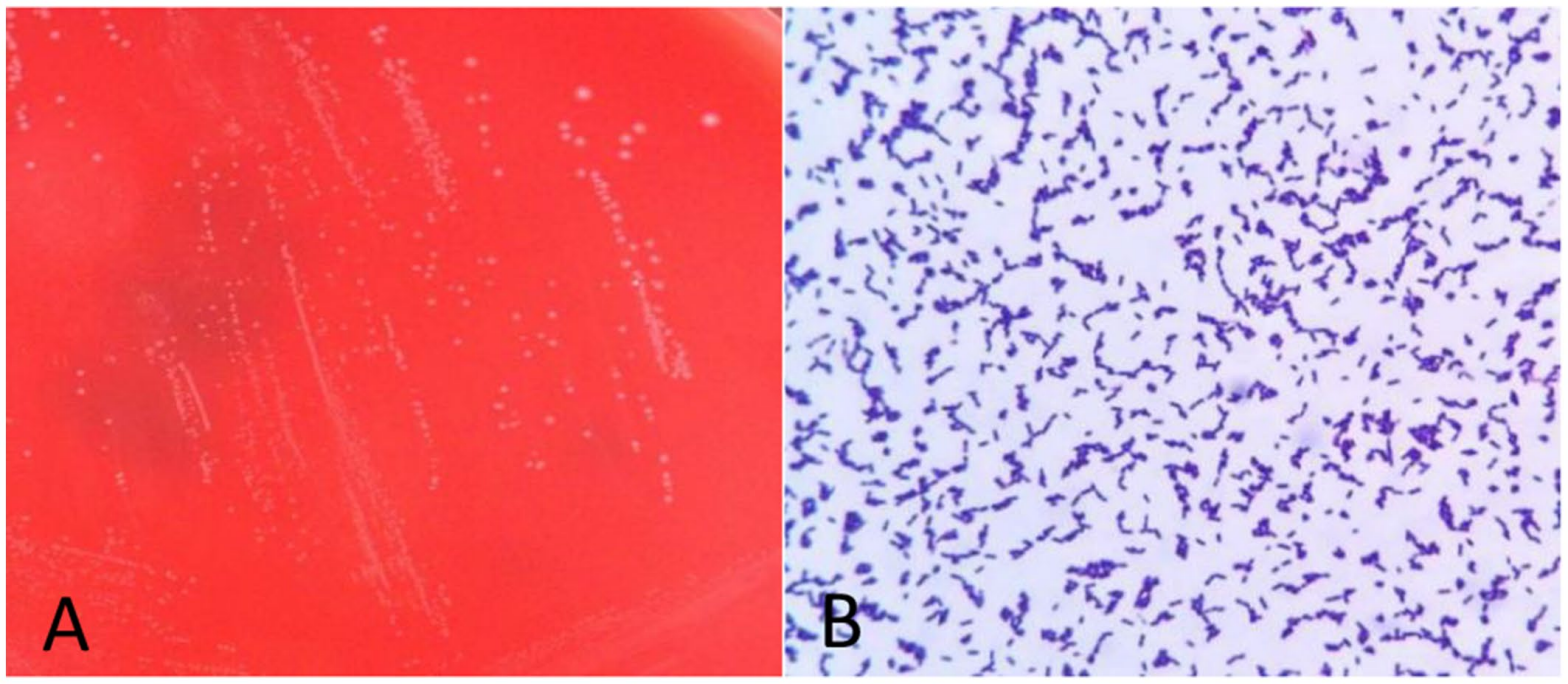
Morphology and gram staining of pure culture colonies of *E. lenta*. **(A)** Columbia blood plate pure fraction colony morphology. **(B)** Gram staining of *C. innocuum*.

Anaerobic blood culture bottle was positive after 20 h of incubating on automatic blood culture system. Positive blood cultures were smeared and stained with gram stain, and gram staining positive bacilli were detected with variable morphology and no typical spores, as shown in Figure 1. Positive blood culture was subcultured on blood plates anaerobically at 37 °C. Twenty-four hours later, colony growth with single morphology was observed macroscopically, which was grayish, flat, rough and irregular in color, and gram staining of single colony smears showed gram-positive bacillus with relatively uniform morphology, as shown in Figure 2A,3, with slight horse stable-like odor. *C. innocuum* (identification score: 9.245) was identified by matter-assisted laser desorption/ionization time of Flight (MALDI-TOF) mass spectrometry (Autof ms 1,000). After 48 h of anaerobic culture, another pinpoint fine colony grew on anaerobic blood plate (Figure 2B). Until 72 h, the colony was about 0.5 mm in diameter, white in color, protruding and neatly marginated (Figure 2C). Gram staining of single colony smear showed morphologically consistent gram-positive bacillus. As shown in Figure 4, MALDI-TOF mass spectrometry identified *E. lenta* (identification score: 9.356). Two kinds of bacteria were purified and cultured anaerobically at 37 °C. E-Test method was used for susceptibility testing of anaerobes. Susceptibility breakpoints for *C. innocuum* and *E. lenta* are not currently available and are used for interpretation of susceptibility results for these two organisms based on susceptibility breakpoints for other anaerobes in 2024 Clinical and Laboratory Standards Institute (CLSI) document, as shown in Table 1, both anaerobes are susceptible to imipenem and tetracycline.

**Table 1:** Susceptibility results for *C. innocuum* and *E. lenta.*

Antimicrobial agents	*C. innocuum*		*E. lenta*	
	MIC	Results	MIC	Results
Clindamycin	> 256	resistant	3	intermediate
Penicillin	0.38	sensitive	2	resistant
Ampicillin	0.19	sensitive	1.5	intermediate
Cefotaxime	1.5	sensitive	256	resistant
Imipenem	3	sensitive	0.5	sensitive
Meropenem	4	sensitive	0.75	sensitive
Tetracycline	0.048	sensitive	0.75	sensitive
Chloramphenicol	32	resistant	3	resistant

In this case, the patient had postoperative history of rectal cancer, poor basic conditions, low immunity, and there was a possibility of infection caused by intestinal colonizers entering the blood, considering that the two anaerobic bacteria in blood culture were pathogenic. According to the literature and drug sensitivity test results of our center, vancomycin 1 g + piperacillin / tazobactam 4.5 g was administered every 12 h for anti-infection for 7 days. The patient’s fever peak decreased, and blood culture turned negative after multiple reexaminations, thus the treatment was effective.

In conclusion, due to the diversity of pathogenic bacteria in the intestinal tract, the cultivation techniques have limitations in terms of nutritional conditions and cannot fully detect all the pathogens. Based on the possibility of other potentially drug-resistant positive bacteria from the abdominal cavity and intestinal tract, vancomycin was used for broad-spectrum bactericidal treatment. According to literature reports (6–8, 22), piperacillin/tazobactam is effective against *C. innocuum* infections. And it has been proven that the combination of vancomycin and piperacillin/tazobactam is indeed effective in treating the patient’s infection.

## Discussion

*innocuum* is an opportunistic pathogen (2, 9, 23). Ha et al. found that *C. innocuum* is a commensal organism of the gut involved in wound healing and lipogenesis that plays an important role in maintaining intestinal health status (24). In immunocompromised patients, *C. innocuum* predisposes to opportunistic infections following dysbacteriosis of the intestinal flora (4). In addition, a large retrospective study of patients with antibiotic-associated diarrhea (AAD) conducted by Taiwanese investigators showed *C. innocuum* as the main cause of extraintestinal *Clostridium* infection (9). In 2022, this team’s AAD case–control study found that *C. innocuum* was more likely to cause extraintestinal *Clostridium* species infections and gastrointestinal symptoms than *C. difficile*. *C. innocuum* infections resulted in a 30-day mortality rate of 14.5% and an overall mortality rate of 23.0% and should be regarded as the causative agent and given timely and correct diagnosis and treatment (23). Since the discovery of *C. innocuum*, extraintestinal *Clostridium* species infections (EIECs) caused by *C. innocuum* have rarely been reported. Existing literature on extraintestinal infections caused by *C. innocuum* is dominated by case reports, most of which are associated with immunocompromised patients or with underlying diseases, and extraintestinal *Clostridium* species infections caused by *C. innocuum* are mostly bacteremia, endocarditis, osteomyelitis, and peritonitis (2). The above studies were similar to this case report. In this case, the patient had multiple metastases of rectal adenocarcinoma and was in a state of extremely low immunity combined with severe underlying disease after 15 cycles of chemotherapy. *C. innocuum* was identified in polymicrobial bloodstream infections after radical colorectal surgery and hepatic lobectomy. Because *C. innocuum* has high resistance to vancomycin and high mortality in immunocompromised patients (8), special attention should be paid to it. It is worth mentioning that *C. innocuum* combined with *E. lenta* causing bloodstream infection after colorectal surgery is rare.

Bacteremia caused by *E. lenta,* an intestinal commensal, is uncommon, with approximately 150 cases reported to date (19). Lee et al. reported the first case of *E. lenta* bloodstream infection in Korea in 2014, similar to the case presented here, and the first Korean patient with *E. lenta* bloodstream infection had previously been diagnosed with rectal cancer and underwent lower gastrointestinal related surgery and chemotherapy (22). The above two cases verified that malignant tumors, history of lower gastrointestinal surgery and immune disorders were predisposing factors for *E. lenta* bloodstream infection. While Acker et al. first reported a case of bloodstream infection with four anaerobic bacteria, *Bacteroides fragilis*, *Bacteriophilus warneri*, and *Neisseria gastroscopici*, which was a patient with hematologic cancer who did not have clear gastrointestinal symptoms, regarding the source of bloodstream infection with these four anaerobic bacteria, the authors posited that it may be associated with oral flora imbalance and intestinal flora translocation caused by long-term hydroxyurea use in patients (19). A study of 16 bacteremic patients showed that overall 60-day mortality was 19% in all cases, and *E. lenta* was detected in the infected bloodstream of patients who died (14). The mortality rate of patients with polymicrobial bloodstream infection ranged from 21 to 63% (8), it has a relatively high mortality rate. In the patient that we have reported here, *C. innocuum* and *E. lenta* were two anaerobic bloodstream infections, and rapid, accurate, and reliable pathogen identification and drug susceptibility testing were important in guiding timely clinical adjustment of anti-anaerobic treatment.

With the development of microbial identification technology, traditional microbiological methods have no advantage in the identification of rare bacteria and fastidious bacteria. MALDI-TOF mass spectrometry, 16S rRNA sequencing, metagenomic next-generation sequencing (mNGS) and other technologies are playing an increasingly important role in the identification of clinical pathogens (25). Direct mass spectrometry identification of positive blood cultures (single pathogen) reduces the secondary reporting time of positive blood cultures to hours or even minutes, but is not suitable for the identification of pathogens of bloodstream co-infections caused by two or more pathogens (26). Due to the complex composition of intestinal flora, when the intestinal mechanical barrier is damaged and local mucosal immune function is impaired, especially in a population with underlying diseases, cancer or immune dysfunction, the possibility of mixed infection of multiple pathogens should be considered when bloodstream infection occurs after colorectal surgery (15). While direct mass spectrometry identification cannot be performed for positive blood cultures of mixed infection (26), 16S rRNA sequencing can accurately identify pathogens, but pure colonies also need to be isolated and cultured, and the types of identification are limited and the cost is high. While mNGS technology can detect various pathogens in specimens without bias, with the advantages of high throughput and full coverage, it is also expensive and cumbersome to operate. In this patient with polymicrobial bloodstream infection, two types of gram-positive bacilli with different morphology were observed by direct smear of positive blood culture. Further isolation and culture were performed. MALDI-TOF mass spectrometry analysis was performed on the pure culture. *C. innocuum* and *E. lenta* were identified at 48 h and 72 h after receiving the samples. The related pathogens were identified more economically and rapidly by traditional culture combined with molecular biology techniques to guide the rational use of drugs in clinical practice and promote the rehabilitation of patients. Therefore, although molecular biology techniques play an increasingly prominent role, traditional smear recognition ability and isolation and culture techniques cannot be ignored. This especially true when mixed bloodstream infection is suspected and similar bacteria are difficult to distinguish (e.g., via bacterial shape, arrangement, and Gram staining of direct smears of positive blood cultures). In such instances, the culture time of transferred blood plates should be appropriately prolonged to increase the detection rate of pathogens with slow growth rate.

In summary, first, when clinicians suspect mixed anaerobic bloodstream infection based on the patient’s clinical symptoms, and when conditions permit, they can conduct both high-throughput metagenomic pathogen sequencing (mNGS) and blood culture tests simultaneously before using antibiotics. mNGS can clearly define the range of pathogens, and promptly alert clinicians to select broad-spectrum antibacterial drugs based on the pathogen spectrum for empirical treatment. At the same time, based on the mNGS results, it is suggested that targeted cultivation of traditional microorganisms be carried out to increase the detection rate and improve the accuracy of mNGS results. This novel report accurately and economically identified *C. innocuum* and *E. lenta*, two rare anaerobic bacteria, using traditional bacterial smear Gram staining and culture methods combined with MALDI-TOF mass spectrometry analysis, thereby avoiding missed diagnosis and reducing the burden on patients. Secondly, for such specimens, the laboratory can extend the blood culture time and use nutrient-rich culture media for appropriate cultivation. Thirdly, once the blood culture test is positive, the staff should carefully examine the direct blood smear of the positive blood culture. Identifying different morphological pathogenic bacteria can facilitate the subsequent solid subculture and the precision of colony identification. Fourth, extending the incubation time and increasing the types of nutrient media and differential media will facilitate the detection of mixed infections.

According to the literature reports and the results of drug sensitivity test performed by our center combined with CLSI on the two anaerobic bacteria, we developed an anti-infective regimen. After treatment, the patient had a flat body temperature and achieved a good therapeutic effect. In conclusion, this article highlights *C. innocuum* and *E. lenta* as rare pathogens of postoperative bloodstream infection in this case of rectal cancer. Given the extremely high mortality rate due to mixed bloodstream infections, timely and appropriate treatment is very important to save the patient’s life.

## Data Availability

The original contributions presented in the study are included in the article/supplementary material, further inquiries can be directed to the corresponding authors.
