# Correction: Case Report: Bloodstream infection due to *Clostridium innocuum* combined with *Eggerthella lenta*

**DOI:** 10.3389/fmed.2026.1823481

**Published:** 2026-03-31

**Authors:** Yan Xu, Anan Xu, Jiaying Du, Yu Zhao, Xiaoqing Liu, Zhewei Sun, Qingqing Xu, Manman Zhang, Yue-ru Tian

**Affiliations:** 1 Department of Laboratory Medicine, Huashan Hospital Fudan University, Shanghai, China; 2 Department of Laboratory Medicine, Liyang People's Hospital, Changzhou, China; 3 Department of Laboratory Medicine, Shidong Hospital, University of Shanghai for Science and Technology, Shanghai, China; 4 Key Laboratory of Clinical Pharmacology of Antibiotics, National Health Commission of the People's Republic of China, Beijing, China; 5 Department of Laboratory Medicine, Jinan Third People's Hospital, Jinan, China

**Keywords:** *Clostridium innocuum*, colorectal surgery, *Eggerthella lenta*, polymicrobial bloodstream infection, rectal cancer

Authors Anan Xu and Jiaying Du were erroneously omitted as equal contributing authors. The statement “These authors share first authorship” has been added to authors Yan Xu, Anan Xu and Jiaying Du.

The original version of this article has been updated.

